# One-Year Period Prevalence of Oral Aphthous Ulcers and Oral Health-Related Quality of Life in Patients with Behçet's Disease

**DOI:** 10.1155/2014/930348

**Published:** 2014-03-09

**Authors:** Mariko Naito, Yoshimi Suzukamo, Kenji Wakai, Miki Azechi, Fumio Kaneko, Takeo Nakayama, Nobuyuki Hamajima, Shunichi Fukuhara

**Affiliations:** ^1^Department of Preventive Medicine, Nagoya University Graduate School of Medicine, 65 Tsurumai-cho, Showa-ku, Nagoya 466-8550, Japan; ^2^Department of Physical Medicine and Rehabilitation, Tohoku University Graduate School of Medicine, 2-1 Seiryo-machi, Aoba-ku, Sendai 980-8575, Japan; ^3^School of Dental Hygiene, Ogaki Women's College, 1-109 Nishinokawa-cho, Ogaki 503-8554, Japan; ^4^Institute of Dermato-Immunology and -Allergy, Southern TOHOKU Research Institute for Neuroscience, 7-115 Yatsuyamada, Koriyama 963-8563, Japan; ^5^Department of Health Informatics, Graduate School of Medicine and Public Health, Kyoto University, Yoshida-Konoe-cho, Sakyo-ku, Kyoto 606-8501, Japan; ^6^Department of Healthcare Administration, Nagoya University Graduate School of Medicine, 65 Tsurumai-cho, Showa-ku, Nagoya 466-8550, Japan; ^7^Department of Epidemiology and Healthcare Research, Graduate School of Medicine and Public Health, Kyoto University, Yoshida-Konoe-cho, Sakyo-ku, Kyoto 606-8501, Japan; ^8^Centre for Innovative Research for Communities and Clinical Excellence (CIRC^2^LE), Fukushima Medical University, 1 Hikariga-oka, Fukushima 960-1295, Japan

## Abstract

The aim of this study was to investigate the 1-year period prevalence of oral aphthous ulcers (OAUs) and their association with oral health-related quality of life (OHQOL) in patients with Behçet's disease (BD) and in the general population. In this cross-sectional study, 675 patients with Behçet's disease (BD group) and 1,097 males and females in the Japanese general population (control group) completed both questionnaires on their OAU status during the prior year and the General Oral Health Assessment Index (GOHAI). In the BD group, 84% of patients reported experiencing an OAU during the previous year, and the mean number of OAUs/year was 13. In the control group, 31% of individuals experienced an OAU during the previous year, and the mean number of OAUs/year was one. Multivariate analysis indicated that both BD patients (OR, 6.2; 95% CI, 4.8–8.0) and controls (OR, 2.6; 95% CI, 2.0–3.5) who had OAUs at least twice per year were more likely to have GOHAI scores below the norm than were controls who had fewer than two OAUs per year. The association between HLA-B∗51 and OAUs remains unknown. The presence of OAUs has a negative effect on the OHQOL of patients with BD.

## 1. Introduction

Behçet's disease (BD) is a multisystem disease characterized by recurrent oral and genital ulcers and relapsing uveitis with mucocutaneous, articular, neurological, urogenital, vascular, intestinal, and pulmonary manifestations. Most patients with BD experience recurrent aphthous stomatitis (RAS), which is often the initial feature of the disease [1]. The prevalence of RAS has been reported to vary between 1% and 66% among adults [2, 3]. BD patients may suffer more RAS events in daily life than do individuals in the general population, although the descriptive data on RAS are insufficient.

Previous studies have detailed the impact of BD, including its symptoms and disease activity, on quality of life (QOL) [4–13]. Blackford et al. [4] found that oral and genital ulcerations in particular affected patients' personal relationships. BD patients with ocular involvement have been reported to be susceptible to anxiety and depression [5]. Arthritis had a significant effect on both the pain level and QOL of patients with BD, leading to an increased risk of mental distress, including anxiety or depression [8].

Oral health status is closely associated with QOL [14]. Tabolli et al. [15] showed that oral mucosal conditions could have a radical effect on oral health-related QOL (OHQOL). Mumcu et al. [9] reported that BD patients with active oral ulcers reported relatively poor OHQOL compared with ulcer-free patients. OHQOL was worse in BD patients than RAS patients, whereas BD patients exhibited a prolonged healing time for oral ulcers, and RAS patients showed an increased frequency of oral ulcers [9].

The extant data are insufficient for clarifying the effects of oral aphthous ulcers (OAUs) on the OHQOL of patients with BD. To our knowledge, no study has analyzed a large dataset including a control group from the general population. Therefore, the aim of this study was to investigate the annual prevalence of OAUs and their association with OHQOL in BD patients and to compare them with those in the general Japanese population.

## 2. Materials and Methods

### 2.1. Study Subjects

Two groups of subjects participated in this study, patients with BD (BD group) and individuals representative of the general population (control group). For the BD group, a cross-sectional study was performed of all members of the Japanese Association for Patients with Behçet's Disease between October 2004 and January 2005. The control group was selected via stratified, multistaged random sampling from 200 locations in Japan. Stratification was performed according to area and municipality based on national census figures. To obtain first-stage sampling units, municipalities were sampled for each stratum. For second-stage sampling, survey units were randomly sampled for each stratum. To obtain third-stage sampling units, individuals from the Basic Resident Registry who were eligible for participation in the survey and resided within the survey areas were systematically sampled at regular intervals. The cross-sectional study was performed in February 2006.

Questionnaires were administered to 2,400 potential control subjects and 1,358 BD patients aged 15–79 years and 17–91 years, respectively. The Ethics Board of iHope International approved this study protocol in October 2004, and the Ethics Board of the Nagoya University School of Medicine approved the study protocol in December 2005.

### 2.2. Instruments

The questionnaire packet included items regarding oral health status, current number of teeth, and the Japanese version of the General Oral Health Assessment Index (GOHAI). The GOHAI contains 12 questions, each of which is scored between 1 and 5; total scores range from 12 to 60—a higher total score indicates a better OHQOL.

The GOHAI, an instrument designed to assess OHQOL, was originally developed in the United States for use among elderly individuals [16]. The Japanese version of the GOHAI questionnaire has been adapted for and validated in the Japanese population [17]. The national norms for Japanese are available online [18]. This index defines health-related QOL as a person's assessment of how the following affects his or her well being: functional factors, psychological factors, social factors, and the experience of pain or discomfort. When the assessment is focused on orofacial concerns, OHQOL is assessed [19].

Patients in the BD group self-reported disease information. They were evaluated for disease severity according to the criteria proposed by the Behçet's Disease Research Committee of Japan in 2003 [20].

### 2.3. Data Analysis

Differences in GOHAI scores and in the frequency and duration of OAUs were analyzed using *t*-tests. For purposes of data analysis, GOHAI scores were treated as dichotomous categorical outcome variables. Group (i.e., control with or without OAUs, BD with or without OAUs) was included as an independent variable. Covariates included age and sex. Age-adjusted and multivariate-adjusted logistic regression models were used to assess the association between BD, experiencing OAUs at least twice per year (“with OAUs” was defined as ≥2 outbreaks during the past year), and the risk of scoring below the national norms on the GOHAI (GOHAI score = 53.1). Responses were analyzed after excluding subjects with missing data related to their OAU 1-year period prevalence, GOHAI scores, or BD diagnosis.

Mean values are reported with standard deviations (SDs). Odds ratios are reported with 95% confidence intervals (95% CIs). All *P*-values were two sided, and *P* < 0.05 was considered to indicate statistical significance. Analyses were performed using the IBM SPSS Statistics software (version 20 for Windows; IBM Corporation, New York, NY, USA).

## 3. Results

### 3.1. Response Rates and Compositions of Final Groups

Responses were obtained from 1,170/2,400 potential control subjects (response rate = 49%) and 883/1358 patients with BD (response rate = 65%). Analyses were limited to data from subjects aged 20–79 years because most respondents (1,122/1,170 controls, 867/883 BD patients) were in this age range. Both sexes were well represented in both groups. The final data analysis was conducted after 192 patients and 25 controls were excluded due to incomplete data. The mean age of the final control group (50.8 ± 15.7 years) differed significantly from that of the final BD group (55.5 ± 12.5 years; *P* < 0.001). The demographic characteristics of the BD group and those of participants in the National Survey of BD patients in 2002 [21] were similar. The mean ages of males were 50.2 and 47.8 years and those of females were 51.3 and 51.3 years in the BD group and the National Survey, respectively. The respective male: female ratios were 1.07 and 0.93.

The mean disease duration for BD patients was 22 ± 12 years (range, 1–50 years). The most common drug treatments used by BD patients were colchicines and steroids. Detailed data on the demographic characteristics of both groups and the disease severity of and drug treatments used by BD patients are presented in Table 1.

### 3.2. Characteristics of the 1-Year Period Prevalence of OAUs

We found that 83.9% of BD patients and 31.0% of controls, regardless of age, had OAUs during the prior year. The mean number of OAUs per year was 12.7 (SD = 31.5) in BD patients and 1.1 (SD = 2.4) in the control group. BD patients experienced OAUs significantly more frequently than did controls (Table 2).

Among patients with BD, more males than females in all age groups, with the exception of 40–59 years, reported having had OAUs. We found a significant difference between males and females in the 1-year period prevalence of OAUs only among those aged 30–39 years (*P* = 0.042). The mean number of persons having OAUs decreased significantly with age among males (*P* = 0.007). In the control group, more females than males in all age groups, except those aged 20–29 and 60–69 years, reported having had OAUs. The mean number of persons having OAUs decreased significantly with age among both males and females (*P* < 0.001). Detailed data on the frequency of OAUs in each group according to sex and age are presented in Table 2.

The mean duration of OAUs was 55.3 ± 76.4 days in the BD group and 4.7 ± 12.1 days in the control group. The duration of OAUs suffered by BD patients was substantially longer than was that experienced by members of the control group, irrespective of sex. The trends in duration for each age category were congruent with the frequency data. Detailed data on the frequency of OAUs in each group by sex and age are provided in Table 2.

Among patients with BD, more females than males in all age groups, with the exception of 30–39 years, reported OAUs of longer duration. We found no significant differences between males and females in all age groups, with the exception of 30–39 years (*t*(60) = 2.520, *P* = 0.014). A similar trend was observed in the control group, with more females than males in all age groups, with the exception of 20–29 years, reporting OAUs of longer duration. We found significant differences in OAU duration between males and females in the 20–29-year-old and 40–49-year-old groups (*t*(107) = 2.078, *P* = 0.040 and *t*(198) = −2.554, *P* = 0.012, resp.).

### 3.3. Effect of OAUs on OHQOL

The 1-year period prevalence of BD had a negative effect on the GOHAI scores of both males and females (both *P* < 0.001). The age-stratified analysis of mean GOHAI scores indicated that the trend observed in females with BD was similar to that in females in the control group. Males without BD had decreased GOHAI scores with age, but those in the 20–39-year-old and 70–79-year-old subgroups of BD patients had lower scores.

After adjusting for age and sex, BD patients with OAUs (where “with OAUs” was defined as ≥2 outbreaks in the last year) were more likely to have GOHAI scores below the norm. Detailed multivariate analysis results before and after stratification for sex and age are reported in Table 3. Stratified analyses by sex and age suggested that the adjusted odds for GOHAI scores below the norm were highest for female BD patients with OAUs who were 60–79 years, followed by female BD patients with OAUs who were 20–39 years. The adjusted odds among control participants with OAUs were higher than were those among BD patients without OAUs.

## 4. Discussion

In the present study, we confirmed that BD patients had a considerably higher frequency of OAUs and a lower OHQOL than the general population. Our sex-stratified analysis further revealed that OHQOL was particularly compromised among females with OAUs. This work corroborates the findings of a prior study indicating that BD patients had lower OHQOL than did healthy controls [9]. The strengths of our study were (1) the inclusion of more BD patients compared with previous studies, (2) the relatively high response rate for a mail-in survey, and (3) the inclusion of a control group drawn from the general population.

The reported 1-year period prevalence rates for RAS vary widely (5–60%), depending on the population examined, with a potential female predominance in adults [22–30]. According to a population study in the United States, only 1% of children had recurrent oral ulcers, but 35–40% may have had a history of RAS-like diseases, with ulceration beginning before 5 years of age [31]. Moreover, the prevalence of affected patients increased with age. However, a study of elderly dental patients in Thailand found RAS in only 0.7% of persons older than 70 years of age [14]. Because OAU status was self-reported in our study, we could not determine whether RAS was one form of BD symptoms. Nevertheless, it was clear that BD patients had a greater burden of suffering related to OAUs than did the general population.

Oral diseases give rise to significant morbidity, resulting in physical, social, and psychological consequences that affect QOL [9]. Oral health problems can result in pain and discomfort and lead to problems with eating, interpersonal relationships, appearance, and self-image. Pain is an important factor that can limit oral functions. Bernabé et al. showed that BD had a considerable impact on QOL as assessed by the generic questionnaire, the EuroQol (EQ-5D) [32].

Al-Omiri and colleagues recently found that patients with RAS reported higher levels of anxiety than did controls. Females (both patients and controls) had higher scores on the Hospital Anxiety and Depression Scale than did males [33]. Stress may trigger the mechanisms involved in the pathogenesis of recurrent OAUs, leading to pain and negative effects on daily activities, initiating a cycle in which stress leads to ulcers and ulcers lead to stress [33, 34]. Pain-related interference appears to be related to psychological rather than to physical characteristics [34]. Krisdapong et al. showed that the discomfort of RAS primarily affected eating and tooth brushing [35]. Our findings show that those in the control group who had OAUs had a lower OHQOL than did BD patients without OAUs. Indeed, recurrent OAUs may have a major negative impact on the OHQOL of not only patients but also of healthy individuals. Prevention of and treatment for OAUs may play an important role in maintaining or improving QOL, particularly in BD patients.

In our study, female BD patients with two or more OAUs within 1 year had almost double the risk for a subnormal GOHAI score than did their male counterparts. This difference should be evaluated further given that it persisted after multivariate adjustment. Gur et al. suggested that BD patients could benefit from psychoeducational or self-management interventions aimed at enhancing self-esteem and ability to manage their disease and its symptoms on a daily basis. They showed that such programs could improve patients' physical and psychological functioning [8].

Although an association between HLA-B*51 and RAS has been reported [36], the findings remain inconsistent [37, 38]. Oral streptococci have been suggested as important determinants of RAS, either as direct pathogens or as an antigenic stimulus culminating in the genesis of antibodies that may cross-react with keratinocyte antigenic determinants [39–41].* Helicobacter pylori* [42, 43], herpes viruses [44–46],* Varicella zoster* virus [47], and cytomegalovirus [47, 48] have also been implicated in RAS. Our study could not analyze the associations between these factors and OAUs because of a lack of information.

This study had several limitations. First, the cross-sectional nature of the data restricts our ability to make inferences regarding the direction of associations. However, it is less reasonable to believe that the domains of QOL affect the 1-year period prevalence OAUs than vice versa. Second, we used a mail-in questionnaire for data collection, and the diagnosis of BD was self-reported. However, we analyzed the data from only those subjects who provided a confirmed year of BD diagnosis. It is unlikely that this practice resulted in misclassifications. Third, this study focused on the frequency of OAUs but not on their severity. Finally, it has been suggested that various physiological and biochemical factors are associated with recurrent aphthous ulcers (e.g., smoking and other lifestyle habits). These factors should be evaluated in greater detail according to the different clinical presentations of the disease.

In conclusion, our data indicate that having OAUs has a negative impact on OHQOL, particularly for female BD patients. Caring for OAUs may be a critical factor in maintaining and improving the QOL of BD patients. Associations between biological features, such as HLA-B*51, and the prevalence of OAUs remain unknown. A more detailed analysis including biological factors may reveal additional associations between OAUs and BD.

## Figures and Tables

**Table 1:** Characteristics of study participants in the final analysis (*n* = 1,772).

Variable	BD patients		Controls	
	*n*	(%)	*n*	(%)
Sex				
Male	349	(51.7)	532	(48.5)
Female	326	(48.3)	565	(51.5)
Age				
20–29	22	(3.3)	109	(9.9)
30–39	62	(9.2)	195	(17.8)
40–49	112	(16.6)	200	(18.2)
50–59	183	(27.1)	231	(21.1)
60–69	212	(31.4)	206	(18.8)
70–79	84	(12.4)	156	(14.2)
Disease severity				
No symptoms	89	(13.2)		
I	283	(41.9)		
II	131	(19.4)		
III	31	(4.6)		
IV	91	(13.5)		
V	17	(2.5)		
Unknown	33	(4.9)		
Drug treatment				
Colchicines	217	(33.8)		
Steroids	201	(31.0)		
Immunosuppressant	56	(8.4)		
Other(s)	152	(23.3)		

**Table 2:** One-year period prevalence, frequency, and mean total duration of OAUs during the past year in the general population (controls) and BD patients according to sex and age (*n* = 1,772).

Sex, age	No. BD patients^a^/no. controls^a^	BD patients					Controls					*P*-value		
		One-year period prevalence, %	Mean no. OAUs	SD	Mean total duration, day	SD	One-year period prevalence, %	Mean no. OAUs	SD	Mean total duration, day	SD	One-year period prevalence	Mean no. OAUs	Mean total duration
Males														
20–29	11/64	81.8	24.4	(30.9)	38.3	(38.6)	54.7	2.8	(4.0)	13.1	(21.7)	0.091	0.043	0.059
30–39	25/93	100.0	32.0	(52.5)	111.0	(104.7)	38.7	1.3	(2.2)	6.5	(14.2)	<0.001	0.007	<0.001
40–49	61/90	90.2	16.1	(47.3)	60.5	(91.0)	25.6	1.1	(2.9)	3.0	(8.5)	<0.001	0.016	<0.001
50–59	102/111	80.4	9.0	(11.8)	47.4	(68.9)	23.4	0.6	(1.6)	2.3	(5.6)	<0.001	<0.001	<0.001
60–69	109/110	67.0	10.2	(35.8)	39.7	(68.1)	16.4	0.6	(2.5)	1.6	(4.8)	<0.001	0.006	<0.001
70–79	41/64	73.2	6.2	(10.4)	34.6	(66.3)	15.6	0.3	(0.9)	1.5	(5.9)	<0.001	0.001	0.003
Total	**349/532**	**78.5**	**12.4**	**(33.1)**	**61.7**	**(77.2)**	**27.8**	**1.0**	**(2.6)**	**4.2**	**(11.5)**	**<0.001**	**<0.001**	**<0.001**
Females														
20–29	11/45	100.0	9.6	(9.5)	47.3	(34.2)	51.1	1.7	(2.7)	6.8	(9.5)	0.003	0.021	0.003
30–39	37/102	94.6	9.3	(10.5)	57.2	(63.3)	49.0	1.7	(2.5)	7.6	(14.2)	<0.001	<0.001	<0.001
40–49	51/110	94.1	18.9	(52.5)	89.2	(88.8)	41.8	1.5	(2.6)	7.4	(15.6)	<0.001	0.022	<0.001
50–59	81/120	91.4	14.4	(26.5)	65.7	(79.8)	30.8	1.0	(2.4)	4.1	(10.9)	<0.001	<0.001	<0.001
60–69	103/96	83.5	8.5	(10.8)	50.0	(75.2)	13.5	0.4	(1.1)	1.5	(5.4)	<0.001	<0.001	<0.001
70–79	43/92	88.4	18.2	(40.7)	57.4	(75.6)	25.0	0.7	(1.8)	4.3	(15.0)	<0.001	0.007	<0.001
Total	**326/565**	**89.6**	**13.0**	**(29.6)**	**61.7**	**(77.2)**	**34.0**	**1.1**	**(2.3)**	**5.2**	**(12.7)**	**<0.001**	**<0.001**	**<0.001**

^a^All BD patients and controls were analyzed, regardless of the 1-year period prevalence of OAUs.

**Table 3:** Odds ratios (with 95% CI) for GOHAI scores lower than Japanese norms based on 1-year period prevalence of OAUs^a^ in the past year for controls and BD patients.

Group^a^	Total			Males			Females		
	No. with GOHAI scores higher than the norm/no. with those lower than the norm	Crude OR (95% CI)	OR^b^ (95% CI)	No. with GOHAI scores higher than the norm/no. with those lower than the norm	Crude OR (95% CI)	OR^c^ (95% CI)	No. with GOHAI scores higher than the norm/no. with those lower than the norm	Crude OR (95% CI)	OR^c^ (95% CI)
All ages									
GP without OAUs	523/319	1	1	268/153	1	1	255/166	1	1
GP with OAUs	107/148	2.3 (1.7–3.0)	2.6 (2.0–3.5)	47/64	2.4 (1.6–3.7)	2.8 (1.8–4.3)	60/84	2.2 (1.5–3.2)	2.6 (1.8–3.9)
BD without OAUs	87/59	1.1 (0.8–1.6)	1.0 (0.7–1.4)	60/38	1.1 (0.7–1.7)	0.9 (0.6–1.4)	27/21	1.2 (0.7–2.2)	1.0 (0.6–1.8)
BD with OAUs	113/416	6.0 (4.7–7.8)	6.2 (4.8–8.0)	73/178	4.3 (3.0–6.0)	4.4 (3.1–6.1)	40/238	9.1 (6.2–13.5)	9.3 (6.4–13.6)
20–39 years									
GP without OAUs	131/55	1	1	69/27	1	1	62/28	1	1
GP with OAUs	65/53	1.9 (1.2–3.1)	1.9 (1.2–3.1)	33/28	2.2 (1.1–4.2)	2.2 (1.1–4.3)	32/25	1.7 (0.9–3.4)	1.7 (0.9–3.4)
BD without OAUs	3/4	3.1 (0.7–14.7)	3.1 (0.7–14.2)	1/1	2.6 (0.2–42.3)	2.7 (0.2–44.6)	2/3	3.3 (0.5–21.0)	3.5 (0.6–22.3)
BD with OAUs	19/58	7.3 (4.0–13.3)	7.5 (4.0–13.8)	10/24	6.1 (2.6–14.5)	6.1 (2.6–14.4)	9/34	8.4 (3.5–19.8)	9.8 (4.1–23.7)
40–59 years									
GP without OAUs	212/123	1	1	112/57	1	1	100/66	1	1
GP with OAUs	35/61	3.0 (1.9–4.8)	2.9 (1.8–4.8)	10/22	4.3 (1.9–9.7)	4.4 (1.9–9.9)	25/39	2.4 (1.3–4.3)	2.5 (1.4–4.6)
BD without OAUs	32/21	1.1 (0.6–2.0)	1.2 (0.7–2.2)	26/13	1.0 (0.5–2.1)	0.9 (0.4–1.9)	6/8	2.0 (0.7–6.1)	1.6 (0.6–4.8)
BD with OAUs	63/179	4.9 (3.4–7.0)	5.0 (3.4–7.1)	41/83	4.0 (2.4–6.5)	4.1 (2.5–6.7)	22/96	6.6 (3.8–11.6)	6.8 (3.9–11.9)
60–79 years									
GP without OAUs	180/141	1	1	87/69	1	1	93/72	1	1
GP with OAUs	7/34	6.2 (2.7–14.4)	6.1 (2.6–14.3)	4/14	4.4 (1.4–14.0)	4.6 (1.4–14.7)	3/20	8.6 (2.5–30.1)	8.2 (2.3–28.9)
BD without OAUs	52/34	0.8 (0.5–1.4)	1.0 (0.6–1.6)	33/24	0.9 (0.5–1.7)	1.0 (0.5–1.8)	19/10	0.7 (0.3–1.6)	0.8 (0.4–1.8)
BD with OAUs	31/179	7.4 (4.7–11.5)	8.3 (5.3–13.0)	22/71	4.1 (2.3–7.2)	4.9 (2.7–8.9)	9/108	15.5 (7.3–32.7)	15.2 (7.5–30.6)

^a^For purposes of analysis, the cutoff for “with OAUs” was ≥2 OAU outbreaks within the last year.

^b^Adjusted for age and sex.

^c^Adjusted for age.
